# Pre- and Postoperative Evaluation of Immediate and Early Implant Placement in Esthetic Areas with Pre-Extraction Facial Dehiscence: A Retrospective Clinical Study

**DOI:** 10.3390/jcm12206616

**Published:** 2023-10-19

**Authors:** Misa Fujii, Tamaki Nakano, Shoichi Ishigaki

**Affiliations:** Department of Fixed Prosthodontics, Osaka University Graduate School of Dentistry, Osaka 565-0871, Japan; fujii.misa.dent@osaka-u.ac.jp (M.F.); ishigaki.shoichi.dent@osaka-u.ac.jp (S.I.)

**Keywords:** dental implant, implant surgery, soft tissue management, immediate implant placement, early implant placement, connective tissue graft

## Abstract

The clinical requirement for a good esthetic result for immediate implant placement is the absence of dehiscence in the anterior facial alveolar bone. In the presence of dehiscence, it is recommended to use a connective tissue graft in addition to immediate implant placement or to change to early implant placement. However, the literature focusing on dehiscence is scarce, and the influence of different placement times and combined use of connective tissue graft on postoperative esthetics in cases with dehiscence is unclear. Therefore, we quantitatively evaluated the pre-extraction dehiscence morphology and postoperative changes in the facial tissue of implants in three groups: immediate implant placement (Group I), immediate implant placement with connective tissue graft (Group IC), and early implant placement (Group E). To this end, 52 implants were obtained (20 in Group I, 16 in Group IC, and 16 in Group E). A wider dehiscence increases the risk of soft tissue regression, which was one reason for choosing early implant placement. A combination of immediate implant placement and connective tissue graft, or early implant placement, tended to result in less soft tissue regression due to the thicker postoperative facial soft tissue volume.

## 1. Introduction

Esthetic implant treatment requires high survival rates and quality esthetic results. Therefore, the timing of implant placement after tooth extraction should be carefully selected. In implant treatment, the appropriate timing of implant placement after extraction in an esthetic single tooth has long been debated [1]. In 2004, the timing of implant placement was classified into immediate implant placement, early implant placement, and delayed implant placement [2].

- Immediate implant placement into extraction sockets is defined as “Type 1” implant placement;
- When the objective is the soft tissue coverage of the extraction socket, then a healing period of 4–8 weeks is observed before implant placement, which is defined as “Type 2” implant placement;
- When the socket is allowed to heal to have a partial bone fill of the socket, typically 12–16 weeks, this is defined as “Type 3” implant placement;
- When the extraction is fully healed after 16 weeks or more, the implant placement procedure is defined as “Type 4”.

In 2008, immediate (Type 1), early (Types 2 and 3), and delayed (Type 4) implant placement protocols presented a comparable survival rate of >95% [3].

Immediate implant placement has been increasingly chosen to shorten the treatment period and reduce the number of surgical procedures [4,5,6]. However, it has been reported that the number of cases is limited due to the risk of postoperative soft tissue regression and aesthetic problems [7,8]. If the application of immediate implant placement is predicted to result in esthetic complications, the use of connective tissue grafting or a change to early implant placement is recommended [1,9,10]. In a recent literature review, facial dehiscence before tooth extraction is considered one factor affecting the aesthetic outcome of immediate implant placement [9,11]. Dehiscence is often observed in the facial alveolar bone of natural teeth scheduled for implant treatment [12,13], but scarce research focuses on the presence of facial dehiscence before tooth extraction. It is unclear whether connective tissue graft or early implant placement affects postoperative esthetic results in cases with the dehiscence of the facial alveolar bone. The reasons may be that the method of dehiscence and the reference point of measurement differ from one report to another and that there is a lack of evaluation of the treatment results. 

The method of evaluating the dehiscence of the facial alveolar bone before tooth extraction has been reported in the previous research, but bone sounding [14,15] and the measurement method of intraoperative dehiscence using a probe [16,17] are less accurate, and it is undeniable that the measurement reference point for dehiscence is unclear. The method of evaluating dehiscence using CBCT (dental cone-beam CT) before surgery [18,19] has high measurement accuracy [20]; however, in these reports, the crest of the palatal side of the extraction socket and the bottom of the nasal cavity (maxillary bone) were used as the measurement reference points. The shape of the extraction fossa considerably varies from case to case, and the maxillary morphology varies from patient to patient, so these points are not necessarily considered appropriate reference points for measurement.

In this study, a method was used to superimpose preoperative and postoperative CBCT image data. By using this method, it is possible to place an implant model with the same morphology as the placed implant at the same position on the preoperative CBCT image data and to measure the preoperative dehiscence using the implant model as a reference, which facilitates the standardization and quantification of the preoperative dehiscence of the facial alveolar bone.

The purpose of this study was to retrospectively evaluate the pre-extraction alveolar bone morphology and postoperative facial tissue morphology of immediate and early placement implant by superimposing pre- and postoperative CBCTs in the esthetic area with facial dehiscence before tooth extraction. 

This study was conducted after obtaining approval by the Ethics Committee of the Osaka University Graduate School of Dentistry and School of Dentistry Hospital (Approval No. R2-E20).

## 2. Materials and Methods

### 2.1. Study Design and Population 

The study included 505 patients with maxillary anterior or premolar implants placed at the Department of Fixed Prosthodontics, Osaka University Graduate School of Dentistry, from August 2011 to June 2019. Patients were included in the study if they met the following criteria:

- Implant placement must be performed on the day of extraction or between 4 and 16 weeks after extraction, at the same time as bone grafting;
- The presence of dehiscence in the facial alveolar bone before tooth extraction;
- An implant body with a tapered joint and platform shifting is inserted;
- A fixed superstructure is set.
- Patients were excluded from the study if they met the following criteria:
- Smoking [21];
- Patients treated for diabetes, including controlled patients [22];
- Patients with an acute infection at the planned extraction of the tooth and periodontal tissue.

Thus, 52 patients (male: 21, female: 31, mean age: 60.0 ± 14.0 years) who met all inclusion criteria were included in this study.

### 2.2. Classification of Target Patients According to Different Surgical Techniques

The 52 patients were assigned to three groups according to the differences in placement implant as follows:

- For the immediate implant placement group, primary surgery was performed on the same day as tooth extraction (Group I, mean age: 61.5 ± 14.0 years, 9 males, 11 females);
- Group IC patients underwent primary surgery on the same day as tooth extraction and connective tissue grafting was also performed (mean age: 58.4 ± 15.2 years, 6 males, 10 females);
- The early implant placement group (Group E, mean age: 61.8 ± 20.0 years, 6 males, 10 females) underwent primary surgery after waiting 4 to 16 weeks for healing after tooth extraction. In Group E, no connective tissue grafting was performed.

### 2.3. Implant Materials

The implants used in this study were taper-jointed implants with platform switching (Nobel Biocare, Switzerland, or Straumann, Switzerland). The implants were 12.2 ± 1.1 mm (10.0 mm–14.0 mm) in mean length and 3.8 ± 0.4 mm (3.3 mm–4.3 mm) in mean diameter.

### 2.4. Surgical Procedure

The implant surgery was performed by three specialists of the Japanese Society of Oral Implantology with more than 10 years of clinical experience.

Implant placement was performed according to the protocol recommended by each implant manufacturer.

### 2.5. Bone Graft Materials

Bone grafting was performed in all cases. We used Bio-Oss (Geistlich, Switzerland) as bone grafting material and Bio-Gide (Geistlich, Switzerland). To maintain the continuity of the proximal, distal, and facial alveolar bone with reference to the adjacent bone morphology, we placed Bio-Oss into the labial side of the implant at the primary surgery and placed a Bio-Gide to cover the placed Bio-Oss.

### 2.6. Prosthetic Procedure

Because of preoperative facial alveolar bone dehiscence, the subjects in this study were repaired after a healing period. In immediate implant placement, a healing cap was fabricated to seal the mucosa, which was allowed to heal for 6 months. In early implant placement, the mucosa was completely sealed after implantation and allowed to heal for 6 months.

After secondary surgery, impressions were taken after the soft tissue had healed. We set a provisional restoration, checked for no problems, and then constructed and set a final restoration.

### 2.7. CBCT Protocol

CBCT was performed using an Alphard 3030 (Asahi X-ray Industries, Kyoto, Japan) (Table 1). The patient’s posture during imaging was seated, and a cotton roll was inserted into the oral vestibule at the implant site before imaging to depict the peri-implant soft tissues so that the superstructure and soft tissues did not come into contact with the lips and buccal mucosa [23].

CBCT was taken at three time points: before tooth extraction (T0), at the time of superstructure placement (T1), and approximately one year after placement (T2).

### 2.8. Accuracy of Superposition of CBCT before and after Operation

**Figure 1:**
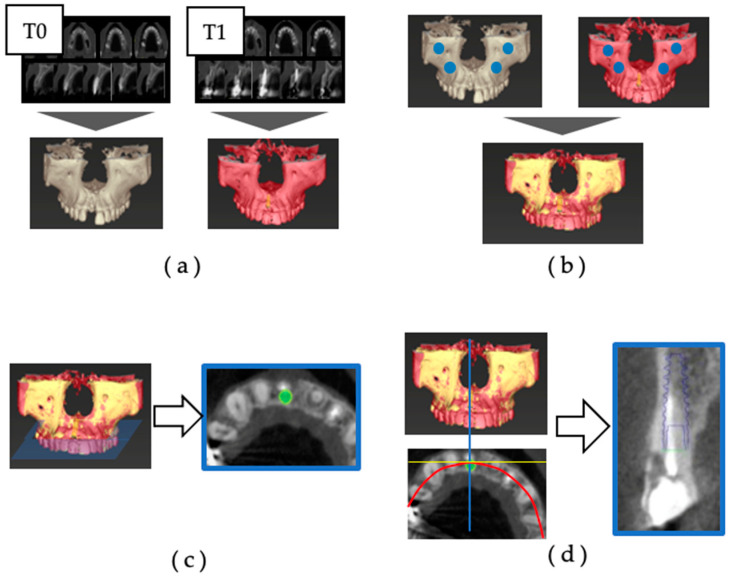
Superimposition of preoperative and postoperative CBCT: (**a**) the 3D jawbone models of T0 and T1 were created based on CBCT data; (**b**) the reference points for superimposition were set at the infraorbital foramen and zygomatic process on the left and right sides, and the 3D jaw models of T0 and T1 were superimposed; (**c**) using the superimposition of the three-dimensional jawbone model, an implant model (IM) was placed in the same position as the actually placed implant body on the preoperative CBCT data. The dental arch was set to pass through the center of the implant body and the center of the remaining teeth on the horizontal section, and the cross-section including the platform of the IM was defined as the axial section; (**d**) in the axial section, the cross-section was defined as the section that was orthogonal to the tangent line of the dental arch and passed through the center of the IM.

Superposition was performed through the following procedure (Figure 1) [24]:

(a) The 3D jawbone models of T0 and T1 were created based on CBCT data.
(b) The reference points for superimposition were set at the infraorbital foramen and zygomatic process on the left and right sides, and the 3D jaw models of T0 and T1 were superimposed.
(c) Using superimposition of the three-dimensional jawbone model, an implant model (IM) was placed in the same position as the actually placed implant body on the preoperative CBCT data. The dental arch was set to pass through the center of the implant body and the center of the remaining teeth on the horizontal section, and the cross-section including the platform of the IM was defined as the axial section.
(d) In the axial section, the cross-section was defined as the section that was orthogonal to the tangent line of the dental arch and passed through the center of the IM.

The facial alveolar bone morphology was evaluated using the acquired axial and cross-sectional images, and the dehiscence was measured with the IM as a reference. Image reconstruction and the measurement of CBCT data were carried out using the digital diagnostic imaging software Co DiagnostiX (latest version 10.7) (Dental Wings, Montreal, QC, Canada).

**Figure 2:**
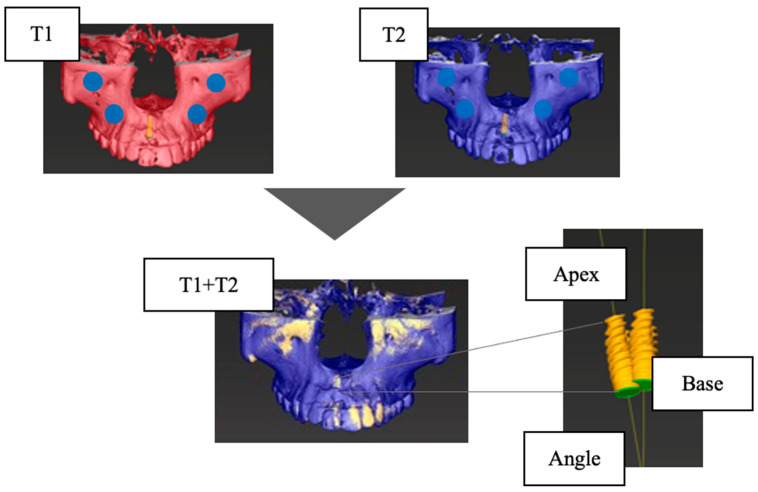
Verification of the accuracy of CBCT superimposition. Base, the distance between the centers of the platform of the implant model (mm); Apex, the distance between the tops of the implant models (mm); Angle, the angle between the long axes of the implant model (°); T1, at the time of superstructure placement; T2, at the time of approximately one year after placement.

The accuracy of the CBCT superimposition was verified by measuring the positional relationship of the IMs on the superimposed jaw models in three dimensions. The measurement items were the distance between the IM platform centers (Base (mm)), the distance between the IM tips (Apex (mm)), and the angle between the IM long axes (Angle (°)) (Figure 2). Then, 10 patients were randomly selected from the 52 patients, and for each of the 10 implants, the T1 and T2 jaw models were superimposed, and the IMs’ positional relationship (Base, Apex, and Angle) was measured. The superimposition of the jaw models and the measurement of the IM positional relationship were performed 5 times for each of the 10 selected patients, and the average values were calculated. The accuracy was high (Table 2). 

### 2.9. Measurement of Facial Alveolar Bone Morphology before Operation

**Figure 3:**
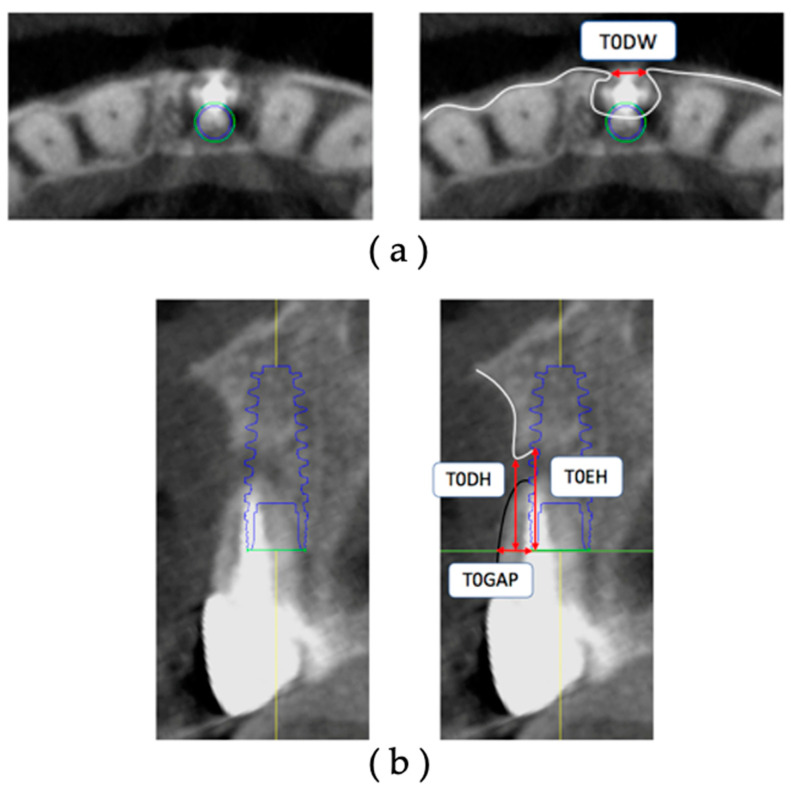
Measurement items for preoperative facial alveolar bone morphology: (**a**) axial view: T0DW, mesial and distal width of dehiscence of the facial alveolar bone; (**b**) cross-sectional view: T0DH, depth of dehiscence of the facial alveolar bone; T0EH, amount of implant exposure; T0GAP, buccolingual width of the tooth root based on the most facial side of the IM.

The CBCT data of T0 were extracted, and the facial alveolar bone morphology before extraction was measured on the diagnostic imaging software (Figure 3). All measurements were performed using the axial section and cross-section acquired with the IM as the reference.

The sites under study were measured on the obtained axial section with respect to the platform level. The measurement sites were the facial alveolar bone dehiscence width (T0DW) on the acquired axial section and the facial alveolar bone dehiscence height (T0DH), the amount of exposure height (T0EH) of the implant body, and the gap width of the tooth root (T0Gap) starting from the most facial side of the IM on the cross-section. All measurements are in millimeters.

Intraexaminer and interexaminer reliability rates in measuring preoperative facial alveolar bone morphology were determined using the interclass correlation coefficient (ICC). One examiner randomly selected 10 patients (10 implants) from the subject patients. Three-dimensional jawbone models of T0 and T1 were created, and both were superimposed on diagnostic imaging software.

Using the superimposition of the 3D jawbone model, the IM was displayed on the CBCT data of T0 at the same position as the actually placed implant body.

T0DW, T0DH, T0EH, and T0GAP were measured on each cross-section. The interval between the first measurement and the second measurement was one week. The intraexaminer reliability was calculated by setting the measurement cross-section and measuring the facial alveolar bone morphology twice before surgery. Similarly, 2 examiners randomly selected 10 implants from the target patients, set the cross-section for measurement, and performed the preoperative measurement of facial alveolar bone morphology once and separately. Both ICCs were high (Table 3), and it was therefore concluded that intra- and interexaminer reliability rates were high for all measurement items.

### 2.10. Measurement of Facial Alveolar Bone Morphology after Restoration

In each group, CBCT data at T1 and T2 were extracted, and the postoperative facial tissue of the implant body was measured on diagnostic imaging software. 

**Figure 4:**
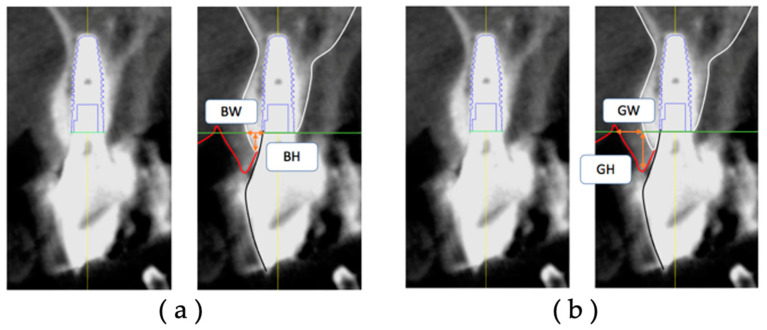
Measurement items for postoperative implant facial tissue based on the platform level: (**a**) BW, the thickness of the hard tissue on the facial side (including bone replacement material) (mm); (**b**) BH, the height of the hard tissue on the facial side (including bone replacement material) (mm); GW, the thickness of the soft tissue on the facial side (mm); GH, the height of the soft tissue on the facial side (mm).

The thickness (BW) and height (BH) of the facial hard tissue of the implant and the thickness (GW) and height (GH) of the facial soft tissue of the implant were measured at T1 and T2, respectively, based on the platform level in the cross-sectional view (Figure 4). The hard tissue change (ΔBW and ΔBH) and soft tissue change (ΔGW and ΔGH) were calculated from T1 to T2.

Intra- and interexaminer reliability rates in measuring facial tissue morphology of postoperative implants were determined according to ICC. Both ICCs were high (Table 4), and it was concluded that intra- and interexaminer reliability rates were high for all measurement items.

### 2.11. Statistical Analysis

One examiner measured each item of the pre-extraction facial alveolar bone morphology and the facial tissue of the implants of the three groups with different placement times and compared each item among the three groups.

For statistical analysis, the Kruskal–Wallis test was used to compare the mean values between the three groups, and the Mann–Whitney U test with Bonferroni correction was used for multiple comparisons of items that showed differences. All analyses were conducted with SPSS using a *p*-value of 0.017 to determine statistical significance.

## 3. Results

### 3.1. Patients

The 52 implants were classified into three groups according to implant placement: Group I had 20 implants, Group IC had 16 implants, and Group E had 16 implants. Table 5 shows the sex ratio, age, and implant placement site for each group. The length, diameter, and shape of the implant used; implant manufacturer; superstructure fixation style; and survival are shown in Table 6. There were no significant differences in the baseline data among the three groups.

### 3.2. Group Evaluation of Dehiscence Morphology of Facial Alveolar Bone before Tooth Extraction

All measurements were based on an IM that matched the type and position of the actual implant. In T0DW, the values for Group E were significantly greater than those of Group I and Group IC (*p*-value = 0.003). No other results showed significant differences among the groups (Table 7).

### 3.3. Group Evaluation of the Change in Postoperative Implant Facial Tissue Morphology over Time

All measurements were based on the actual implant. The degree of change for each measurement was calculated from T1 to T2 (1-year interval).

In the facial hard tissue of the postoperative implants, ΔBH was significantly greater in Group I than in Group IC and Group E. Other results were not significantly different among the groups (Table 8).

T1GW and T2GW were significantly greater in the facial soft tissues of the postoperative implants in Group IC and Group E than in Group I. ΔGH was significantly lower in Group IC and Group E than in Group I. Other results showed no significant differences among the groups (Table 8). 

**Figure 5:**
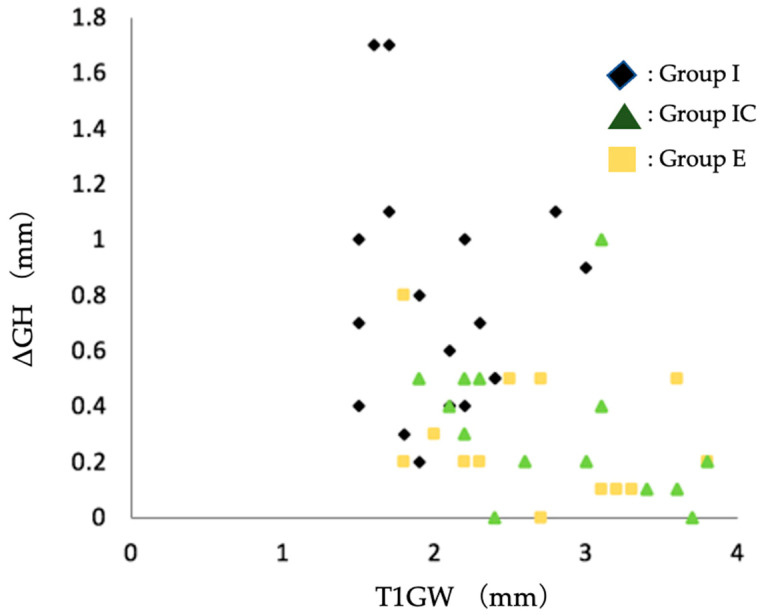
Scatter plot of soft tissue regression (ΔGH) and facial soft tissue thickness at T1. Group I, immediate implant placement group; Group IC, immediate implant placement combined with connective tissue graft group; Group E, early implant placement group; GW, the thickness of the soft tissue on the facial side (mm); ΔGH, the amount of the change in height of the soft tissue from T1 to T2; T1, the time the superstructure was set.

It has been reported that the thickness of the facial soft tissue of postoperative implants affects the amount of soft tissue regression [25]. Therefore, scatter plots of T1GW and ΔGH were drawn and are shown in Figure 5. All but one case in Group IC at T1 and three cases in Group E at T1 had a facial soft tissue thickness of 2 mm or greater, and no case had soft tissue regression of 1 mm or greater.

## 4. Discussion

### 4.1. Group Evaluation of Dehiscence Morphology of Labial Alveolar Bone before Tooth Extraction

Pre-extraction facial alveolar bone morphology was evaluated using superimposition of pre- and postextraction CBCT image data. The quantitative evaluation of CBCT image data is well known in implant treatment, and its accuracy and reproducibility have not been problematic [26]. However, when the IM is located in the same position as the placed implant in the CBCT image data, there is a possibility that some errors may occur due to metallic artifacts of the implant itself. In this method, we measured the error caused by the superposition of CBCT image data taken at different times. The errors were all less than 0.2 mm, and although the values obtained were uncertain because the voxel values of the CBCT images were 0.2 mm, they were highly accurate (Figure 2). Intra- and interexaminer reliability values were considered excellent, with intraclass correlation coefficients exceeding 0.8 for all measurement items (Table 3). In addition to the accuracy of the measurements, the IM was used to set a reference point for the measurements, which was difficult to set using conventional methods. This technique, which enables the evaluation of facial alveolar bone morphology on CBCT images before tooth extraction, may contribute to preoperative diagnosis to determine implant placement.

**Figure 6:**
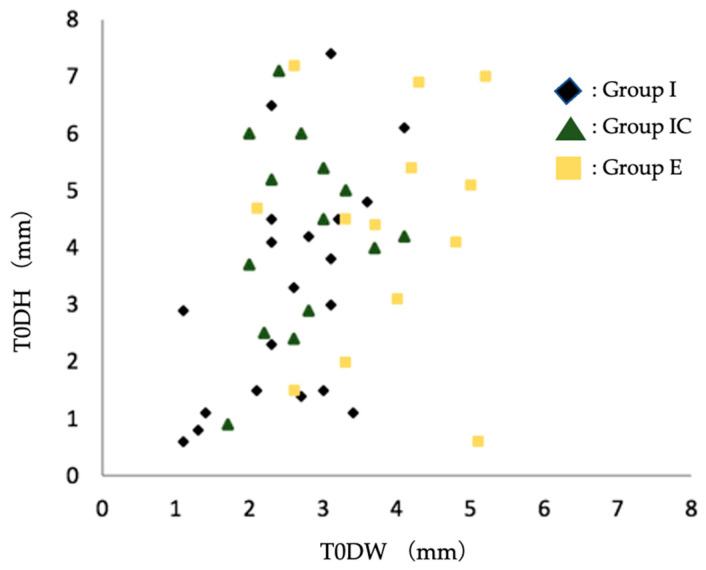
Scatter plot of preoperative facial alveolar bone morphology in terms of dehiscence height (DH) and dehiscence width (DW). Group I, immediate implant placement group; Group IC, immediate implant placement combined with connective tissue graft group; Group E, early implant placement group; T0DW, mesial and distal width of dehiscence of the facial alveolar bone; T0DH, depth of dehiscence of the facial alveolar bone.

Among the dehiscence morphology of the facial alveolar bone before extraction, the width of the dehiscence morphology was significantly larger in Group E than in Group I and Group IC. Based on the scatter plots of dehiscence width and depth, there was a tendency toward the selection of early implant placement when the width of the dehiscence exceeded 3–4 mm (Figure 6). 

**Figure 7:**
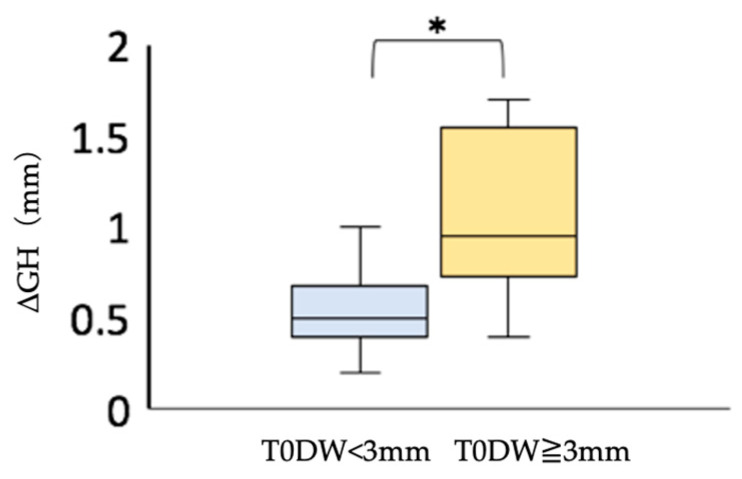
Comparison of postoperative soft tissue regression (ΔGH) in the immediate implant placement group. * Mann–Whitney U test; *p <* 0.05. T0DW < 3 mm, immediate implant placement group with facial alveolar dehiscence of less than 3 mm in width before extraction; T0DW ≧ 3 mm, immediate implant placement group, where the width of the dehiscence in the facial alveolar bone before extraction was 3 mm or more; ΔGH, amount of the change in height of the soft tissue from T1 to T2.

When there is a large dehiscence in the facial alveolar bone where the implant is placed, the probability of postoperative soft tissue regression is reported to be high when immediate implant placement is applied [25]. Among Group I, we compared the postoperative soft tissue regression by dividing the width of the dehiscence into two groups, namely those with a dehiscence of more than 3 mm and those with a dehiscence of less than 3 mm, using Mann–Whitney’s U test with the significance level set at α = 0.05. We found that the postoperative soft tissue regression was significantly greater in the group with a dehiscence size of more than 3 mm (Figure 7). If the facial alveolar bone at the proposed implant placement site had a dehiscence wider than 3 mm based on preoperative CBCT images, soft tissue regression was more likely to be observed postoperatively. Therefore, the presence of a dehiscence wider than 3 mm may be one of the reasons not to choose immediate implant placement. 

### 4.2. Group Evaluation of the Change in Postoperative Implant Labial Tissue Morphology over Time

In this study, osteogenesis was combined in all cases extracted. Bio-Oss is a heterogeneous bone composed of bovine bone, with high porosity [27] and excellent bone conduction ability [28]. Also, although generally classified as non-resorbable, previous reports have revealed that, despite the low rate of resorption, 30% will be replaced by autogenous bone at 8 months postoperatively and 70% at 20 months [28]. It has been reported that Bio-Oss grafts are replaced by autogenous bone to some extent, albeit slowly [29]. When measuring the hard tissues, the distinction between autogenous bone and bone grafts was unclear on the CBCT image data, and the hard tissues of the two types were measured collectively as hard tissues.

Soft tissue measurements include intraoral photographs taken with a digital camera [30] and using models [31]. In this study, we utilized a technique devised in a previous study in which a cotton roll was inserted into the oral vestibule, and CBCT imaging was performed to avoid contact between the facial soft tissue of the implant fixture and the lips [24]. On CBCT images, we could easily distinguish the boundaries between the implant and the alveolar bone, the alveolar bone and natural teeth, the alveolar bone and soft tissues such as gingiva, and soft tissues and air, which have significantly different radiopaque properties. Using this method, the facial soft tissue of the implant in the CBCT image data is clearly depicted. Therefore, soft tissue could be measured using CBCT image data as well as hard tissue.

The postoperative facial tissue morphology of Group I, Group IC, and Group E were compared. The thickness of the facial soft tissue was significantly greater in Group I than in Group IC and Group E at the time of superstructure placement and approximately one year after superstructure placement. Bone loss and soft tissue regression were significantly lower in Group IC and Group E than in Group I (Table 8). It has been reported that, compared with thin soft tissue, the thick soft tissue on the facial side of the implant suppresses tissue regression over time [25,32]. This study showed that immediate implant placement combined with connective tissue grafting resulted in a postoperative facial soft tissue thickness of more than 2 mm (Figure 5). In other words, even if immediate implant placement is applied to a patient with dehiscence in the facial alveolar bone before tooth extraction, connective tissue grafting may help to obtain thicker facial soft tissue after surgery and prevent vertical tissue regression.

The advantages of early implant placement are that if the facial alveolar bone is thin before extraction, the soft tissue volume is increased by waiting for soft tissue healing after extraction [33], and the extraction socket is covered with soft tissue at the time of implant placement, thus obtaining a keratinized gingival width of 3–5 mm [1,16]. In this study, Group E gained almost 2 mm or more of facial soft tissue volume. No more than 1 mm soft tissue regression was observed postoperatively (Figure 5). The results suggest that early implant placement in the facial alveolar bone before extraction in the presence of dehiscence can also provide a thicker soft tissue volume, thus reducing the amount of postoperative vertical tissue regression. 

These results suggest that the combination of immediate implant placement and connective tissue grafting or early implant placement in esthetic areas with pre-extraction dehiscence may reduce the amount of soft tissue regression. 

Postoperative soft tissue regression was less than 1 mm in Group I, with dehiscence less than 3 mm wide (Figure 7). The results suggest that immediate implant placement may provide a good esthetic result when the width of the dehiscence is less than 3 mm.

Finally, in this study, patients were divided into three groups according to treatment technique. Although there were no significant differences in baseline data such as sex ratio, age, or tooth type, the presence of confounding by these factors or unknown factors cannot be ruled out. To prevent these confounding effects, the number of subjects should be increased and further statistical analyses should be conducted in the future.

## 5. Conclusions

We evaluated the pre-extraction dehiscence morphology and postoperative facial tissue morphology of implants with immediate and early placement in esthetic areas where there was a pre-extraction dehiscence morphology in the facial alveolar bone, and we drew the following conclusions:

- It was found that immediate implant placement might have good esthetic results if the dehiscence was less than 3 mm wide. If the dehiscence was wider than 3–4 mm, there was a tendency to avoid immediate implant placement.
- In cases with dehiscence, thicker labial soft tissues could be obtained by combining immediate implant placement and connective tissue grafting or by waiting for the soft tissues to heal after tooth extraction in early implant placement. This result suggests that the amount of soft tissue regression can be reduced.

## Figures and Tables

**Table 1:** CBCT imaging conditions of patients.

Field of View (FOV)	833 cm^3^: Diameter 102 mm × Height 102 mm
Voxel value	0.2 mm
Tube Voltage	80 kV
Tube current	7 mA
Shooting time	17,000 msec

**Table 2:** Verification of the accuracy of CBCT superimposition. Base, the distance between the centers of the platform of the implant model (mm); Apex, the distance between the tops of the implant models (mm); Angle, the angle between the long axes of the implant model (°).

	Average	SD
Base (mm)	0.05	0.02
Apex (mm)	0.07	0.03
Angle (°)	0.21	0.07

**Table 3:** Intra- and interexaminer reliability of preoperative measurement of facial alveolar bone morphology.

Measurement Item	Intraexaminer Reliability ICC (1, 1)	Interexaminer Reliability ICC (2, 1)
T0DW	0.91	0.90
T0DH	0.89	0.87
T0GAP	0.87	0.89
T0EH	0.93	0.94

T0DW, mesial and distal width of dehiscence of the facial alveolar bone; T0DH, depth of dehiscence of the facial alveolar bone; T0EH, amount of implant exposure; T0GAP, buccolingual width of the tooth root based on the most facial side of the IM.

**Table 4:** Intra- and interexaminer reliability of postoperative implant facial tissue measurements.

Measurement Item	Intraexaminer Reliability ICC (1, 1)	Interexaminer Reliability ICC (2, 1)
BW	0.92	0.93
BH	0.87	0.89
GW	0.89	0.87
GH	0.84	0.89

BW, the thickness of the hard tissue on the facial side (including bone replacement material) (mm); BH, the height of the hard tissue on the facial side (including bone replacement material) (mm); GW, the thickness of the soft tissue on the facial side (mm); GH, the height of the hard and soft tissues on the facial side (mm).

**Table 5:** Comparison of baseline data between Group I, Group IC, and Group E (1).

		Group I	Group IC	Group E	*p* Value
Male/female ratio *		9/11	6/10	6/10	0.97
Age (year) ^†^		61.5 ± 13.9	58.4 ± 15.1	61.2 ± 19.3	0.62
Measurement area (location) *	Middle incisors	7	8	5	0.11
	Lateral incisors	9	4	4	
	Canine	2	2	3	
	First premolar	2	2	4	

* Fischer’s exact probability test (significance level α = 0.05); ^†^ Student’s *t*-test (significance level α = 0.05). Group I, immediate implant placement group; Group IC, immediate implant placement combined with connective tissue graft group; Group E, early implant placement group.

**Table 6:** Comparison of baseline data between Group I, Group IC, and Group E (2).

			Group I	Group IC	Group E	*p* Value
Length of implant body (mm) ^†^			12.3 ± 1.1	12.4 ± 1.1	11.8 ± 1.0	0.35
Diameter of the implant body (mm) ^†^			3.7 ± 0.4	4.0 ± 0.4	3.9 ± 0.4	0.11
Manufacturer of implant body, Implant design *	Nobel Biocare	Nobel Active	13	10	7	0.83
		Nobel Parallel Conical Connection	0	1	2	
		Nobel Replace Conical Connection	2	2	2	
	Straumann	BLT	5	3	5	
Superstructure fixation style *	Screw retain		16	12	13	0.98
	Cement retain		4	4	3	
Number of surviving implants			20/20	16/16	16/16	

* Fischer’s exact probability test (significance level α = 0.05); ^†^ Student’s *t*-test (significance level α = 0.05). Group I, immediate implant placement group; Group IC, immediate implant placement combined with connective tissue graft group; Group E, early implant placement group; T0, the time before tooth extraction; T1, the time the superstructure was set; T2, the time of regular recall appointment one year after the superstructure was set.

**Table 7:** Comparison of preoperative facial alveolar bone morphology.

	Group I (*n* = 20)		Group IC (*n* = 16)		Group E (*n* = 16)		*p*-Value *
Measurement Item	Mean (SD)	Median [Max, Min]	Mean (SD)	Median [Max, Min]	Mean (SD)	Median [Max, Min]	
T0DW (mm)	2.5 (0.8)	2.7 [4.1, 1.1]	2.7 (0.7)	2.7 [4.1, 1.7]	3.9 (1.0)	4.0 [5.2, 2.1]	0.003
T0DH (mm)	3.3 (2.0)	3.2 [7.4, 0.6]	4.3 (1.7)	4.4 [7.1, 0.9]	4.3 (2.1)	4.5 [7.2, 0.6]	0.193
T0GAP (mm)	2.1 (0.7)	2.1 [4.7, 0.9]	2.4 (1.1)	2.2 [4.4, 0.6]	2.4 (0.8)	2.5 [4.2, 1.0]	0.531
T0EH (mm)	4.9 (1.5)	5.2 [7.4, 2.0]	5.1 (1.8)	5.6 [7.2, 0.8]	5.4 (2.3)	4.9 [9.6, 2.3]	0.881

* Kruskal–Wallis test, Mann–Whitney U test, Bonferroni correction; *p* < 0.017. Group I, immediate implant placement group; Group IC, immediate implant placement combined with connective tissue graft group; Group E, early implant placement group; T0DW, mesial and distal width of dehiscence of the facial alveolar bone; T0DH, depth of dehiscence of the facial alveolar bone; T0EH, amount of implant exposure; T0GAP, buccolingual width of the tooth root based on the most facial side of the IM.

**Table 8:** Comparison of postoperative implant body facial tissue morphology.

	Group I (*n* = 20)		Group IC (*n* = 16)		Group E (*n* = 16)		*p*-Value *
Measurement Item	Mean (SD)	Median [Max, Min]	Mean (SD)	Median [Max, Min]	Mean (SD)	Median [Max, Min]	
T1BW (mm)	2.1 (0.8)	1.9 [ 3.5, 0.4]	2.0 (1.0)	1.8 [4.0, 0.4]	2.3 (1.0)	2.5 [3.5, 0.6]	0.552
T2BW (mm)	1.6 (0.8)	1.7 [3.4, 0.3]	1.8 (1.0)	1.7 [3.6, 0.4]	2.0 (1.0)	2.0 [3.4, 0.4]	0.459
ΔBW(mm)	0.5 (0.5)	0.3 [2.0, 0]	0.3 (0.2)	0.3 [0.6, 0]	0.3 (0.2)	0.3 [0.8, 0]	0.668
T1BH (mm)	1.7 (0.6)	1.7 [2.8, 0.6]	1.6 (1.0)	1.7 [3.1, 0]	1.6 (0.8)	1.5 [2.7, 0]	0.923
T2BH (mm)	1.0 (0.5)	1.0 [2.1, 0.3]	1.3 (1.0)	1.4 [2.9, 0]	1.3 (0.8)	1.1 [2.6, 0]	0.447
ΔBH (mm)	0.7 (0.4)	0.7 [1.4, 0.2]	0.3 (0.2)	0.2 [0.7, 0]	0.2 (0.2)	0.2 [0.7, 0]	0.000
T1GW (mm)	2.1 (0.4)	2.1 [3.0, 1.5]	2.8 (0.7)	2.8 [3.8, 1.9]	2.7 (0.7)	2.7 [3.8, 1.8]	0.001
T2GW (mm)	1.8 (0.4)	1.8 [2.7, 1.2]	2.6 (0.7)	2.5 [3.7, 1.5]	2.4 (0.6)	2.3 [3.4, 1.5]	0.001
ΔGW (mm)	0.3 (0.2)	0.3 [0.8, 0]	0.2 (0.2)	0.2 [0.7, 0]	0.3 (0.2)	0.2 [0.8, 0]	0.582
T1GH (mm)	4.5 (0.9)	4.4 [5.7, 1.8]	5.0 (1.0)	5.2 [6.2, 2.5]	4.6 (1.0)	4.9 [6.0, 2.9]	0.334
T2GH (mm)	3.8 (1.0)	3.9 [5.2, 1.1]	4.6 (1.0)	4.6 [5.8, 2.1]	4.4 (1.1)	4.1 [6.0, 2.4]	0.054
ΔGH (mm)	0.7 (0.4)	0.6 [1.7, 0.2]	0.3 (0.3)	0.3 [1.0, 0]	0.3 (0.2)	0.2 [0.8, 0]	0.000

* Kruskal–Wallis test, Mann–Whitney U test, Bonferroni correction; *p* < 0.017. Group I, immediate implant placement group; Group IC, immediate implant placement combined with connective tissue graft group; Group E, early implant placement group; BW, the thickness of the hard tissue on the facial side (including bone replacement material) (mm); BH, the height of the hard tissue on the facial side (including bone replacement material) (mm); GW, the thickness of the soft tissue on the facial side (mm); GH, the height of the soft tissue on the facial side (mm); ΔBW, the amount of the change in thickness of the hard tissue from T1 to T2; ΔBH, the amount of the change in the height of the hard tissue from T1 to T2; ΔGW, the amount of the change in thickness of the soft tissue from T1 to T2; ΔGH, the amount of the change in height of the soft tissue from T1 to T2; T1, the time the superstructure was set; T2, the time of regular recall appointment one year after the superstructure was set.

## Data Availability

The datasets used and analyzed during the current study are available from the corresponding author upon reasonable request.
